# Atlas of Venereal and Skin Diseases

**Published:** 1888-04

**Authors:** 


**Atlas of Venereal and Skin Diseases.** By PRINCE A. MORROW, A.M., M.D., Clinical Professor of Venereal Diseases, formerly Clinical Lecturer on Dermatology in the University of the City of New York; Surgeon to Charity Hospital, etc. Wm. Wood & Co., 1888; J. H. Matteson, 16 East Eagle street, Buffalo.

Wm. Wood & Co. have furnished to the profession a great many valuable works, and they assume the most important and expensive undertakings with a boldness founded on years of successful experience. In the preparation of this Atlas, they have enlisted the co-operation of the leading dermatologists and syphilographers of the world. Prominent among them are Profs. M. Kaposi and I. Neumann, of Vienna. The atlas of the former, of venereal diseases, just completed, and that of the latter, of skin diseases, now being issued in parts, will be largely drawn upon in the preparation of this work, the sole right to reproduce them having been obtained by this enterprising firm. Among other distinguished gentlemen who have engaged to contribute selections from their collections of original illustrations, may be mentioned: Dr. J. Hutchinson, of London; Profs. A. Fournier and A. Hardy; and Drs. Ricord, Cullerrier, Besnier, and Vidal, of Paris; Prof. Leloir, of Lille; Dr. P. A. Morrow, of New York; Dr. E. L. Keyes, of New York; Dr. Fessenden N. Otis, of New York; Dr. J. Nevins Hyde, of Chicago; Dr. Henry G. Piffard, of New York, and others. The author of the work is Dr. Prince A. Morrow, of New York, who, in addition to plates contributed from his own remarkable collection, will write the treatise on skin and venereal diseases, which constitutes, besides the description of the plates, the text accompanying them. Particular attention is given to the common forms of skin diseases, and included in it, not only as a valuable independent feature, but also as a practical means of differential diagnosis, are the eruptive fevers, as rubeola, scarlatina, erysipelas, variola, varicella-vaccinia, etc., not found in any other work of the kind. The publishers announce that the "Atlas of Venereal and Skin Diseases" will be published in fifteen imperial folio parts, at the very moderate price of $2.00 a part. The entire work will contain seventy-five superb colored plates, executed in true chromo-lithographic style, exquisitely printed in flesh tints and colors, containing several hundred figures, many life-size, together with descriptive text for each plate, and from sixteen to twenty folio pages of a practical treatise upon venereal and skin diseases; the whole forming one magnificent thick imperial folio volume. We have received the first two parts, and we have only to say that in these the promises made in the announcements of the work are splendidly fulfilled. This Atlas bids fair to be the most superb work of the kind in medical literature.

